# Diabetes and Covid-19 among hospitalized patients in Saudi Arabia: a single-centre retrospective study

**DOI:** 10.1186/s12933-020-01184-4

**Published:** 2020-12-05

**Authors:** Abdullah M. Alguwaihes, Mohammed E. Al-Sofiani, Maram Megdad, Sakhar S. Albader, Mohammad H. Alsari, Ali Alelayan, Saad H. Alzahrani, Shaun Sabico, Nasser M. Al-Daghri, Anwar A. Jammah

**Affiliations:** 1 Division of Endocrinology, Department of Internal Medicine, College of Medicine, King Saud University, Riyadh, 11472 Saudi Arabia; 2 Division of Endocrinology, Diabetes and Metabolism, the Johns Hopkins University, Baltimore, MD 21218 USA; 3 Strategic Center for Diabetes Research, College of Medicine, King Saud University, Riyadh, 11481 Saudi Arabia; 4 General Directorate of Health Affairs in Eastern Province, Dammam, 32247 Saudi Arabia; 5 King Saud University Medical City, Riyadh, 12746 Saudi Arabia; 6 Obesity, Endocrine, and Metabolism Center, King Fahad Medical City, Riyadh, 11564 Saudi Arabia; 7 Chair for Biomarkers of Chronic Diseases, Biochemistry Department, College of Science, King Saud University, Riyadh, 11451 Saudi Arabia

**Keywords:** Diabetes mellitus, Covid-19, Mortality, Saudi Arabia

## Abstract

**Background:**

Information on the clinical characteristics and outcomes of hospitalized Covid-19 patients with or without diabetes mellitus (DM) is limited in the Arab region. This study aims to fill this gap.

**Methods:**

In this single-center retrospective study, medical records of hospitalized adults with confirmed Covid-19 [RT-PCR positive for SARS-CoV2] at King Saud University Medical City (KSUMC)-King Khaled University Hospital (KKUH), Riyadh, Saudi Arabia from May to July 2020 were analyzed. Clinical, radiological and serological information, as well as outcomes were recorded and analyzed.

**Results:**

A total of 439 patients were included (median age 55 years; 68.3% men). The most prevalent comorbidities were vitamin D deficiency (74.7%), DM (68.3%), hypertension (42.6%) and obesity (42.2%). During hospitalization, 77 out of the 439 patients (17.5%) died. DM patients have a significantly higher death rate (20.5% versus 12.3%; p = 0.04) and lower survival time (p = 0.016) than non-DM. Multivariate cox proportional hazards regression model revealed that age [Hazards ratio, HR 3.0 (95% confidence interval, CI 1.7–5.3); p < 0.001], congestive heart failure [adjusted HR 3.5 (CI 1.4–8.3); p = 0.006], smoking [adjusted HR 5.8 (CI 2.0–17.2); p < 0.001], β-blocker use [adjusted HR 1.7 (CI 1.0–2.9); p = 0.04], bilateral lung infiltrates [adjusted HR 1.9 (CI 1.1–3.3); p = 0.02], creatinine > 90 µmol/l [adjusted HR 2.1 (CI 1.3–3.5); p = 0.004] and 25(OH)D < 12.5 nmol/l [adjusted HR 7.0 (CI 1.7–28.2); p = 0.007] were significant predictors of mortality among hospitalized Covid-19 patients. Random blood glucose ≥ 11.1 mmol/l was significantly associated with intensive care admission [adjusted HR 1.5 (CI 1.0–2.2); p = 0.04], as well as smoking, β-blocker use, neutrophil > 7.5, creatinine > 90 µmol/l and alanine aminotransferase > 65U/l.

**Conclusion:**

The prevalence of DM is high among hospitalized Covid-19 patients in Riyadh, Saudi Arabia. While DM patients have a higher mortality rate than their non-DM counterparts, other factors such as old age, congestive heart failure, smoking, β-blocker use, presence of bilateral lung infiltrates, elevated creatinine and severe vitamin D deficiency, appear to be more significant predictors of fatal outcome. Patients with acute metabolic dysfunctions, including hyperglycemia on admission are more likely to receive intensive care.

## Background

The severe acute respiratory syndrome coronavirus 2 (SARS-CoV2) was first identified last December 2019 from a cluster of Wuhan residents initially diagnosed with pneumonia of unknown origin in Hubei, China [1]. As the year 2020 progressed, this novel coronavirus strain became responsible for the catastrophic spread of coronavirus disease-19 (Covid-19), the pandemic which has so far claimed ~ 1.3 million human lives in more than 227 countries and territories as of November, 2020 [2]. Despite the alarming figures, Covid-19 has a fatality rate of 2.3%, much lower than similar outbreaks such as SARS-CoV in 2003 (9.5%) and the Middle East Respiratory Syndrome (MERS) CoV in 2012 (34.4%) [3]. The Kingdom of Saudi Arabia (KSA), which is the largest sovereign state in the Arabian Peninsula in terms of geography and economy, was not only the epicenter of the MERS-CoV outbreak, it is also the most affected country with the highest mortality rate from Covid-19 in the Gulf Cooperation Council (GCC) region, and third highest number of confirmed cases in the Middle East after Iran and Iraq [2]. The kingdom’s capital, Riyadh, has the highest number of SARS-CoV2 infected residents and citizens in the country [4].

In terms of predictors, preliminary evidence from 191 SARS-CoV2 infected patients in Wuhan showed that the most common factors associated with poor prognosis include advanced age, the presence of pre-existing conditions such as hypertension and diabetes mellitus (DM), as well as elevated inflammatory markers, amongst others [5]. These findings have been mostly consistent in other countries [6–8]. In KSA, the earliest national data (March, 2020) involving 1519 confirmed cases (mean age 36 years) also showed that the most common comorbidities were hypertension (8.8%) and DM (7.6%) [9]. This build-up of epidemiologic evidence made it increasingly clear that DM and other chronic, non-communicable diseases appear to negatively influence Covid-19 clinical outcomes [10, 11]. Furthermore, given the coronaviruses’ affinity to angiotensin converting enzyme 2 (ACE2), concerns on the use of anti-hypertensive drugs such as angiotensin receptor blockers (ARBs), β-blockers, calcium channel blockers (CCBs) and ACE inhibitors have been raised, before being deemed safe and in some instances, protective against Covid-19 [12, 13]. ARB (valsartan) in particular, in combination with a neprilysin inhibitor (sacubitril), has been shown in patients to significantly reduce death from cardiovascular causes or first hospitalization for worsening heart failure and death from any cause [14]. Such treatment was more effective in reducing proteinuria, preserving renal ultrastructure and diminishing tubular injury, at least in animal models with early diabetic nephropathy [15].

As more cases have exponentially emerged over the past couple of months and given the novelty of the SARS-Cov2 pathogen, there is a need to update and increase the limited evidence on the ever-changing Covid-19 demographics, particularly in underrepresented regions such as the Middle East and KSA in particular, where chronic, non-communicable diseases such as DM are common [16]. Since most published evidence in this emerging field mostly came from the Far East and Western regions, data from other geographical areas representing other ethnicities, with different healthcare systems, may offer unique perspectives on this on-going pandemic. Indeed, while it has been already observed that DM and other chronic conditions increase morbidity and mortality from Covid-19 [17], individuals from other ethnic minorities have been disproportionately affected [18]. To date, there is very limited evidence on the clinical characteristics and outcomes of hospitalized Covid-19 patients with or without DM in the Middle East and the GCC region in particular. The present study aims to fill this gap.

## Methodology

### Study design and setting—single-center, retrospective. KSUMC, Riyadh, KSA

#### Participants

Records of 439 adult Saudis and residents of Riyadh, KSA, who were confirmed positive for SARS-CoV2 and admitted at KSUMC-KKUH from May to July 2020, were included in this retrospective study. Children, pregnant women and those who tested negative for SARS-CoV2 were excluded. Diagnosis of SARS-CoV2 infection was based on the guidelines set by the Saudi Center for Disease Prevention and Control [19]. In brief, swab samples were obtained from the patient’s upper respiratory tract (nasopharyngeal and/or oropharyngeal), placed in a sterile tube containing viral transport media and delivered immediately to a Biosafety Level 2-facility (BSL-2) with Biological Safety Cabinet Class II (BSC-II) in KSUMC, Riyadh, KSA, for reverse transcription-polymerase chain reaction (RT-PCR) analysis. Sample investigations were performed by certified laboratory personnel following manufacturer’s recommendations for the defined cut-off cycle threshold (CT) value for each target gene. Waiver of informed consent approval was obtained from the Institutional Review Board (IRB) of the College of Medicine in King Saud University in Riyadh, Saudi Arabia (E-20-5090/July 5, 2020).

### Data collection

Clinical information included demographics, symptoms and vital signs on arrival to ER, medical history and list of medications taken. Anthropometrics, serological tests done (complete blood count, profiles of liver, renal, thyroid, lipids, inflammatory markers and others) and chest X-ray findings, if available, were obtained. Management given as well as the number of days from diagnosis to hospital admission, intensive care treatment, intubation and final outcome (discharged, died), were noted. For the purpose of this study, DM was defined as having one or more of the following criteria: known case of DM based on medical records, on anti-DM medications, HbA1c ≥ 6.5 and fasting glucose ≥ 7.0 mmol/l. Non-DM was defined as having one or more of the following: no history of DM and/or anti-DM medication use, HbA1c < 6.5 and fasting glucose < 7.0 mmol/l. Normal ranges of all parameters were based on the cut-offs used in the central laboratory of KSUMC-KKUH where all analysis was done. A patient was considered ‘severe’ if he/she required intensive care on admission.

### Data analysis

Data analysis was done using SPSS version 21.0 (IBM, SPSS, Chicago, IL, USA) Demographic characteristics were presented as percentages (%) and continuous variables were presented as mean ± standard deviation (SD) for normal variables and mean ± standard error mean (SEM) for non-normal variables. Chi Square test was done to determine differences in categorical variables. Independent T-test and Mann–Whitney U-test were done to determine differences between sex (adjusted for age and BMI) and DM status (adjusted for age, sex and BMI) for normal and non-normal variables, respectively. The same tests were applied to determine differences according to severity and final outcome, with Bonferroni adjusted p-values for multiple comparisons. Management and outcomes for all patients as well as significant predictors for each outcome were plotted as figures using MS Excel. Univariate and multivariate Cox-Regression analysis was used to determine unadjusted and adjusted hazard ratios (HR) with 95% confidence intervals (CI) for outcomes. To determine significant pre-existing conditions leading to outcomes of interest, all comorbidities were included in the model, including age, sex and BMI. All other parameters (medications and laboratory investigations) were adjusted for age, sex and BMI. Survival curve for DM was done using Kaplan–Meier and compared using log-rank test. Significance was set at p < 0.05.

## Results

### Patient characteristics and comorbidities

Table 1 shows the demographic and general characteristics of 439 Covid-19 patients admitted, stratified according to sex. The over-all median age was 55 years (minimum 19, maximum 101)]. Female patients were significantly older and had significantly higher BMI than males (p-values 0.03 and 0.001, respectively). Male patients outnumber females 2:1. Saudis represented almost half of all admitted patients (49.7%), with a higher proportion of males than females regardless of nationality (p < 0001). Non-Saudi Arabs were the second most common demographic (22.8%), followed by Indians (11.2%). Three-fourths of all admitted patients whose vitamin D status were assessed had vitamin D deficiency (25(OH)D < 50 nmol/l) (74.7%). Other common comorbidities noted were hypertension (42.6%) and obesity (42.2%), all of which more prevalent in female than male patients (p-values 0.001 and < 0.001, respectively). The prevalence of DM was 68.3% based on known records + newly diagnosed cases as defined previously. Smoking was the least common risk factor with only 2.6% and were mostly male patients (p = 0.05). Among the maintenance medications noted, oral hypoglycemic drugs were the most common (33.7%), followed by statins (25.5%), CCBs (21.8%), anti-platelets (18.2%) and β-Blockers (16.6%). With the exception of ACE inhibitors, female patients had a significantly higher percentage of maintenance medication use than males, including ARBs, CCBs, insulin, oral hypoglycemic and levothyroxine (p-values < 0.001, 0.03, < 0.001, 0.006, 0.04 and 0.001, respectively). None of the admitted patients had COPD.

**Table 1:** General characteristics of Covid-19 Patients

Parameters	All	Males	Females	p-value
N (%)	439 (100)	300 (68.3)	139 (31.7)	
Age (years) Median (min–max)	55 (19–101)	54 (19–87)	59 (20–101)	0.03
BMI (kg/m^2^)	29.7 ± 6.7	28.8 ± 5.8	31.5 ± 8.0	0.001
Nationality (%)				
Saudi	218 (49.7)	123 (41.0)	95 (68.3)	< 0.001
Arab (non-Saudi)	100 (22.8)	73 (24.3)	27 (19.4)	
Filipino	20 (4.6)	16 (5.3)	4 (2.9)	
Bangladeshi	15 (3.4)	15 (5.0)	0	
Pakistani	12 (2.7)	10 (3.3)	2 (1.4)	
Indian	49 (11.2)	47 (15.7)	2 (1.4)	
Afghani	4 (0.9)	3 (1.0)	1 (0.7)	
Others	21 (4.8)	12 (4.3)	8 (5.8)	
Comorbidities (%)				
Obesity	178 (42.2)	101 (35.3)	77 (56.6)	< 0.001
Hypertension	187 (42.6)	109 (36.3)	78 (56.1)	< 0.001
Diabetes mellitus*	300 (68.3)	200 (66.7)	100 (71.9)	NS
Cardiovascular disease	44 (10.0)	31 (10.3)	13 (9.4)	NS
Congestive heart failure	18 (4.1)	12 (4.0)	6 (4.3)	NS
Chronic kidney disease	22 (5.0)	13 (4.3)	9 (6.5)	NS
Stroke	17 (3.9)	10 (3.3)	7 (5.0)	NS
Smoking	9 (2.6)	7 (3.2)	2 (1.6)	0.05
Vitamin D deficiency**	112 (74.7)	75 (75.8)	37 (72.5)	NS
Medications (%)				
β-Blockers	73 (16.6)	50 (16.7)	23 (16.9)	NS
ACE inhibitors	48 (10.9)	33 (11.0)	15 (10.8)	NS
ARB	63 (14.4)	31 (10.3)	32 (23.5)	< 0.001
CCB	95 (21.8)	55 (18.3)	40 (29.4)	0.03
Statins	111 (25.5)	69 (23.1)	42 (30.9)	NS
GLP-1 agonists	5 (1.1)	3 (1.0)	2 (1.5)	NS
Insulin	63 (14.4)	28 (9.4)	35 (25.7)	< 0.001
Oral hypoglycemic	148 (33.7)	89 (29.8)	59 (43.4)	0.006
Anti-coagulants	19 (4.3)	9 (3.0)	10 (7.4)	0.04
Anti-platelets	80 (18.2)	51 (17.0)	29 (21.3)	NS
Levothyroxine	25 (5.7)	9 (3.0)	16 (11.6)	0.001

*DM cases were known + newly diagnosed; **Only 150 cases had vitamin D status; significant at p < 0.05

### Symptoms on admission

Table 2 shows the presenting symptoms and vital signs of all patients on admission. Majority had fever (75.2%), dyspnea (72.8%) and cough (70.0%) on presentation. One out of every 5 patients also had nausea/vomiting (23.1%) and/or diarrhea (21.3%). A small percentage of patients reported anosmia (4.6%), ageusia (5.3%) and myalgia (9.5%). Stratified according to sex, male patients had a significantly higher prevalence of fever and diarrhea than females (p-values 0.01 and 0.04, respectively). Among the vital signs assessed, tachypnea and hypoxemia were observed, as indicated by the over-all mean respiratory rate (25.4 ± 8.9 breaths per minute) and SpO_2_ (91.0 ± 8.7). Respiratory rate was worse in males than females, after adjusting for age and BMI (p = 0.01).

**Table 2:** Presenting symptoms and vital signs of Covid-19 patients on admission

Parameters	All	Males	Females	p-value	Non-DM	DM	p-value
N	439	300	139		139	300	
Symptoms							
Fever (%)	330 (75.2)	236 (80.3)	94 (68.6)	0.01	97 (70.8)	233 (77.7)	NS
Cough (%)	303 (70.0)	208 (70.3)	95 (69.3)	NS	83 (60.1)	220 (74.6)	0.002
Dyspnea (%)	316 (72.8)	224 (75.7)	92 (66.7)	NS	83 (60.1)	233 (78.7)	< 0.001
Nausea/vomiting (%)	100 (23.1)	68 (23.0)	32 (23.4)	NS	29 (21.0)	71 (24.1)	NS
Diarrhea (%)	92 (21.3)	71 (24.1)	21 (15.3)	0.04	33 (24.1)	59 (20.0)	NS
Anosmia (%)	20 (4.6)	11 (3.7)	9 (6.5)	NS	9 (6.5)	11 (3.7)	NS
Ageusia (%)	23 (5.3)	15 (5.1)	8 (5.8)	NS	6 (4.3)	17 (5.7)	NS
Myalgia (%)	41 (9.5)	28 (9.3)	13 (9.4)	NS	14 (10.2)	27 (9.1)	NS
Vital signs*							
Temperature (°C)	37.6 ± 0.8	37.6 ± 0.9	37.5 ± 0.8	NS	37.5 ± 0.8	37.6 ± 0.9	NS
Heart rate (beats/minute)	96.6 ± 17.9	96.4 ± 17.4	97.1 ± 19.1	NS	97.8 ± 17.6	96.1 ± 18.1	NS
Respiratory rate (breaths/min)	25.4 ± 8.9	26.1 ± 9.8	23.9 ± 6.4	0.01	23.8 ± 6.4	26.1 ± 9.8	0.009
Systolic blood pressure (mmHg)	125.0 ± 19.9	125.7 ± 18.8	123.7 ± 22.4	NS	120.8 ± 16.3	127.0 ± 21.2	0.02
Diastolic blood pressure (mmHg)	73.2 ± 13.2	74.0 ± 12.2	71.4 ± 15.1	NS	72.8 ± 11.9	73.4 ± 13.8	NS
SpO_2_ (%)	91.0 ± 8.7	90.8 ± 9.2	91.6 ± 7.4	NS	92.8 ± 7.9	90.2 ± 8.9	NS

*Denotes p-values adjusted for age and BMI in males and females; age, sex and BMI in non-DM and DM; significant at p < 0.05

When stratified according to DM status, DM patients had a significantly higher prevalence of cough and dyspnea than non-DM patients (p-values 0.02 and < 0.001, respectively). In terms of vital signs, tachypnea was significantly worse in DM than non-DM patients, after adjusting for age, sex and BMI (p-value 0.009). Systolic blood pressure was also significantly higher in DM than non-DM patients (p = 0.018). The rest of the parameters are shown in Table 2.

No differences in symptoms were observed among severe (N = 123) and non-severe (N = 316) patients, as well as those who died (N = 77) versus those discharged (N = 343). Concerning vital signs, a significantly lower respiratory rate and SpO_2_ were noted among severe than non-severe cases (p-values < 0.01). Patients who died also had a significantly lower SpO_2_ as well diastolic blood pressure, with a significantly higher respiratory rate on admission than patients who were discharged (p-values < 0.01) (Additional file 1).

### Radiologic and serologic characteristics

Table 3 shows the clinical characteristics of Covid-19 patients based on chest X-ray and laboratory investigations. Majority of patients (60%) showed bilateral lung infiltrates and only 10% showed none. Altered levels of metabolic and inflammatory profiles were observed in almost all serologic tests conducted. Age- and BMI-adjusted differences revealed that male patients had significantly higher hemoglobin and neutrophil counts than females (p-values < 0.001 and 0.04, respectively), while females had a significantly higher platelet count than males (p = 0.04). In the liver profile, male patients also had a significantly higher circulating levels LDH than females (p = 0.003). No differences were observed between sexes with respect to renal profile. Nonetheless, mean circulating BUN and creatinine levels were above normal. Lipid and thyroid profiles were unremarkable. On the other hand, inflammatory markers were markedly elevated in all patients, with males having significantly higher levels of ferritin and CRP (p-values < 0.001 and 0.003) than females. Mean corrected calcium levels were within normal range, but females had significantly higher levels than males (p = 0.02). In all patients, only 150 had records for 25(OH)D levels, majority of whom (74.7%) were well below the deficiency range (not shown in table). Seven patients had severe vitamin D deficiency (< 12.5 nmol/l) (not shown in table). The rest of the parameters were shown in Table 3.

**Table 3:** Clinical characteristics of Covid-19 patients on admission

Parameters	All	Males	Females	p-value*	Non-DM	DM	p-value**
N	439	300	139		139	300	
Chest X-ray No infiltrates	131 (30.4)	94 (31.8)	37 (27.4)	NS	61 (43.9)	70 (23.5)	< 0.001
Unilateral infiltrates	43 (10.0)	31 (10.5)	12 (8.9)		7 (5.3)	36 (12.1)	
Bilateral infiltrates	257 (59.6)	171 (57.8)	86 (61.9)		65 (48.9)	192 (64.4)	
Complete blood count							
Hemoglobin (g/l) (120–160)	130 ± 1.2	134.6 ± 1.4	120.4 ± 1.7	< 0.001	131.2 ± 2.2	129.6 ± 1.3	NS
WBC count (4.0–11.0)	8.0 ± 0.2	8.3 ± 0.3	7.3 ± 0.3	NS	8.0 ± 0.4	8.0 ± 0.3	NS
Platelet count (140–450)	249.7 ± 4.7	244.7 ± 5.7	260.5 ± 8.3	0.04	249.6 ± 8.4	249.7 ± 5.7	NS
Lymphocyte (1–5)	1.3 ± 0.1	1.2 ± 0.1	1.4 ± 0.1	NS	1.5 ± 0.2	1.2 ± 0.1	NS
Neutrophils (2.0–7.5)	6.0 ± 0.2	6.4 ± 0.3	5.3 ± 0.3	0.04	5.8 ± 0.3	6.1 ± 0.3	NS
d-dimer (µg/ml) (0.22–0.45)	2.2 ± 0.2	2.3 ± 0.2	2.0 ± 0.2	NS	1.7 ± 0.2	2.4 ± 0.2	NS
Liver profile							
ALT (U/l) (20–65)	57.6 ± 3.1	62.3 ± 3.2	47.1 ± 7.1	NS	64.7 ± 5.8	54.4 ± 3.7	NS
AST (U/l) (15–37)	60.8 ± 3.1	62.5 ± 2.9	57.0 ± 7.4	NS	68.1 ± 6.9	57.6 ± 3.2	NS
LDH (U/l) (84–246)	455.2 ± 11.9	475.3 ± 14.7	409.0 ± 19.5	0.003	440.2 ± 26.4	461.7 ± 12.7	NS
Renal Profile							
BUN (mmol/l) (2.5–6.4)	7.7 ± 0.4	7.8 ± 0.5	7.4 ± 0.6	NS	6.6 ± 0.7	8.2 ± 0.5	NS
Creatinine (µmol/l) (49–90)	123.6 ± 8.0	129.6 ± 9.8	110.6 ± 13.8	NS	110.4 ± 12.3	129.7 ± 10.2	NS
Na (mmol/l) (136–145)	136.8 ± 0.3	137.0 ± 0.3	136.3 ± 0.5	NS	137.8 ± 0.5	136.4 ± 0.3	NS
K (mmol/l) (3.5–5.1)	4.4 ± 0.1	4.4 ± 0.05	4.6 ± 0.3	NS	4.2 ± 0.1	4.6 ± 0.1	NS
Lipid profile							
Triglycerides (mmol/l)	2.0 ± 0.09	2.1 ± 0.1	1.9 ± 0.1	NS	1.6 ± 0.1	2.1 ± 0.1	0.03
HDL-Cholesterol (mmol/l)	0.88 ± 0.05	0.8 ± 0.1	0.9 ± 0.05	NS	0.8 ± 0.05	0.9 ± 0.06	NS
LDL-Cholesterol (mmol/l)	2.0 ± 0.08	2.0 ± 0.1	2.0 ± 0.1	NS	2.0 ± 0.2	2.0 ± 0.09	NS
Inflammatory markers							
Ferritin (µg/ml) (13–150)	942.6 ± 54.6	1116.3 ± 72.7	570.7 ± 60.9	< 0.001	859.2 ± 90.5	980.7 ± 67.9	0.049
Procalcitonin (ng/ml) (0–0.046)	2.6 ± 0.6	2.0 ± 0.6	3.9 ± 1.5	NS	4.2 ± 1.8	2.0 ± 0.6	NS
ESR (mm/h) (0–24)	70.5 ± 2.0	67.5 ± 2.3	77.2 ± 3.6	NS	63.1 ± 3.8	74.0 ± 2.2	NS
CRP (mg/l) (< 10.0)	104.8 ± 3.8	110.6 ± 4.8	91.2 ± 5.7	0.003	97.3 ± 7.3	107.9 ± 4.4	NS
IL-6 (pg/ml) (1.5–7.0)	172.6 ± 28.3	183.5 ± 37.2	145.4 ± 34.7	NS	143.5 ± 42.3	183.7 ± 35.6	NS
Thyroid profile							
TSH (µIU/ml) (0.25–5.0)	2.1 ± 0.5	2.1 ± 0.7	2.1 ± 0.5	NS	1.7 ± 0.2	2.2 ± 0.7	NS
FT4 (pmol/l) (10–24.5)	16.4 ± 0.3	16.4 ± 0.3	16.5 ± 0.6	NS	15.6 ± 0.5	16.8 ± 0.4	0.02
Glycemic profile							
HbA1c (%)	8.0 ± 0.1	7.9 ± 0.2	8.1 ± 0.2	NS	5.5 ± 0.1	10.6 ± 0.3	< 0.001
Fasting Glucose (mmol/l)	9.2 ± 0.3	9.1 ± 0.3	9.6 ± 0.5	NS	5.8 ± 0.05	8.6 ± 0.1	< 0.001
Other markers							
Corrected Ca (mmol/l) (2.1–2.55)	2.3 ± 0.01	2.31 ± 0.01	2.34 ± 0.01	0.02	2.3 ± 0.01	2.3 ± 0.01	NS
25(OH)D (nmol/l) (75–250)	40.4 ± 2.4	39.0 ± 2.8	43.1 ± 4.4	NS	38.1 ± 6.2	41.1 ± 2.5	NS

*Denotes p-values adjusted for age and BMI; **denotes p-values adjusted for age, sex and BMI; significant at p < 0.05

When stratified according to DM status, age-, sex- and BMI-adjusted comparisons revealed higher prevalence of bilateral lung infiltrates in DM as compared to non-DM patients (p < 0.001). No significant differences observed in most serologic parameters with the exception of triglycerides, ferritin and FT4, all of which were significantly higher in DM than non-DM patients (p-values 0.03, 0.049, 0.014 and 0.02, respectively) (Table 3).

When grouped according to severity status, the presence of bilateral lung infiltrates, mean d-dimer, AST, LDH, ferritin and CRP were all significantly higher among severe than non-severe patients (adjusted p-values < 0.01). Lastly, when measured parameters were compared according to final outcome, patients who died had a significantly higher prevalence of bilateral lung infiltrates as well as significantly higher circulating levels of hemoglobin, neutrophils, d-dimer, BUN, creatinine, potassium, triglycerides, ferritin and CRP on admission, than patients who were discharged (adjusted p-values < 0.01) (Additional file 2).

### Management and outcomes

**Fig. 1:**
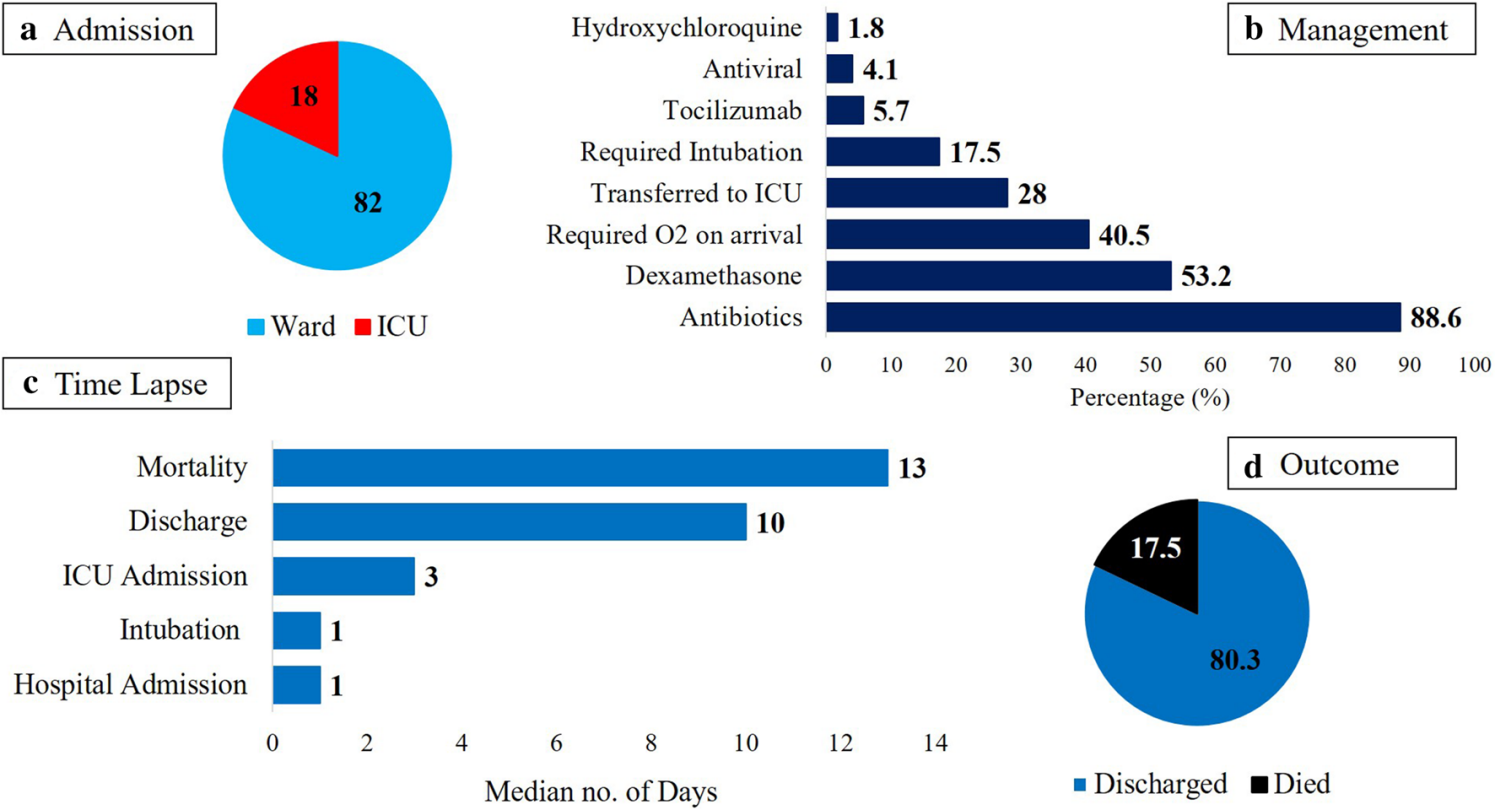
Management and outcomes of all admitted Covid-19 patients (N = 439); **a** Percentage (%) of patients admitted to ward and ICU, **b** Percentage (%) of patients who received (X) intervention, **c** Median number of days from confirmed Sars-CoV2 diagnosis to (X) event and **d** Final outcome of patients

Figure 1 shows that more than 80% of all admitted patients were transferred to ward, while 79 patients (18.0%) needed immediate intensive care. Among the supportive medications provided, antibiotics was the most common (88.6%) and hydroxychloroquine the least (1.8%). During the course of admission, 77 patients (17.5%) required intubation and an additional 123 patients were eventually transferred to ICU. Over-all mortality rate was 17.5% and 80.3% were considered recovered and discharged. Median number of days from diagnosis to ICU admission was 3, from diagnosis to discharge was 10 days and from diagnosis to death was 13 days (Fig. 1). No significant differences were seen in the management and outcomes when stratified according to sex, with the exception of antibiotic use, being modestly more prevalent in males than females (p = 0.05) (Table 4).

**Table 4:** Management and outcomes of Covid-19 patients according to sex and DM status

Parameters (%)	Males	Females	p-value	Non-DM	DM	p-value
N	300	139		139	300	
On admission			NS			NS
Ward	242 (80.7)	118 (84.9)		118 (84.9)	242 (80.7)	
ICU	58 (19.3)	21 (15.1)		21 (15.1)	58 (19.3)	
Required O_2_ on arrival to ER	124 (41.3)	54 (38.8)	NS	43 (30.9)	135 (45.0)	0.006
Antiviral	10 (3.3)	8 (5.8)	NS	9 (6.5)	9 (3.0)	NS
Tocilizumab	19 (6.3)	6 (4.3)	NS	4 (2.9)	21 (7.0)	NS
Hydroxychloroquine	4 (1.3)	4 (2.9)	NS	5 (3.6)	3 (1.0)	NS
Antibiotics	272 (90.7)	117 (84.2)	0.05	106 (76.3)	283 (94.3)	< 0.001
Dexamethasone	164 (54.7)	69 (50.0)	NS	50 (36.2)	183 (61.0)	< 0.001
Required Intubation	54 (18.2)	23 (16.5)	NS	17 (12.3)	60 (20.3)	0.04
Transferred to ICU	82 (27.3)	41 (29.7)	NS	32 (23.4)	91 (30.5)	NS
Outcome						
Mortality	55 (18.6)	22 (16.3)	NS	17 (12.3)	60 (20.5)	0.04
Discharged/Recovered	234 (79.9)	109 (81.3)	NS	118 (86.1)	225 (77.6)	NS
Time lapse from diagnosis [median number of days (min–max)]						
Admission	1 (1–37)	1 (1–32)	NS	1 (1–19)	1 (1–37)	NS
Intubation	1 (1–23)	1 (1–19)	NS	1 (1–20)	1 (1–23)	NS
ICU admission	2 (1–20)	3 (1–16)	NS	3.0 (1–16)	3 (1–20)	NS
Discharge	10 (1–50)	11 (1–50)	NS	10 (2–50)	10 (1–50)	NS
Mortality	13.5 (1–47)	11 (1–29)	NS	15 (1–28)	12 (1–47)	NS

Significant at p < 0.05

**Fig. 2:**
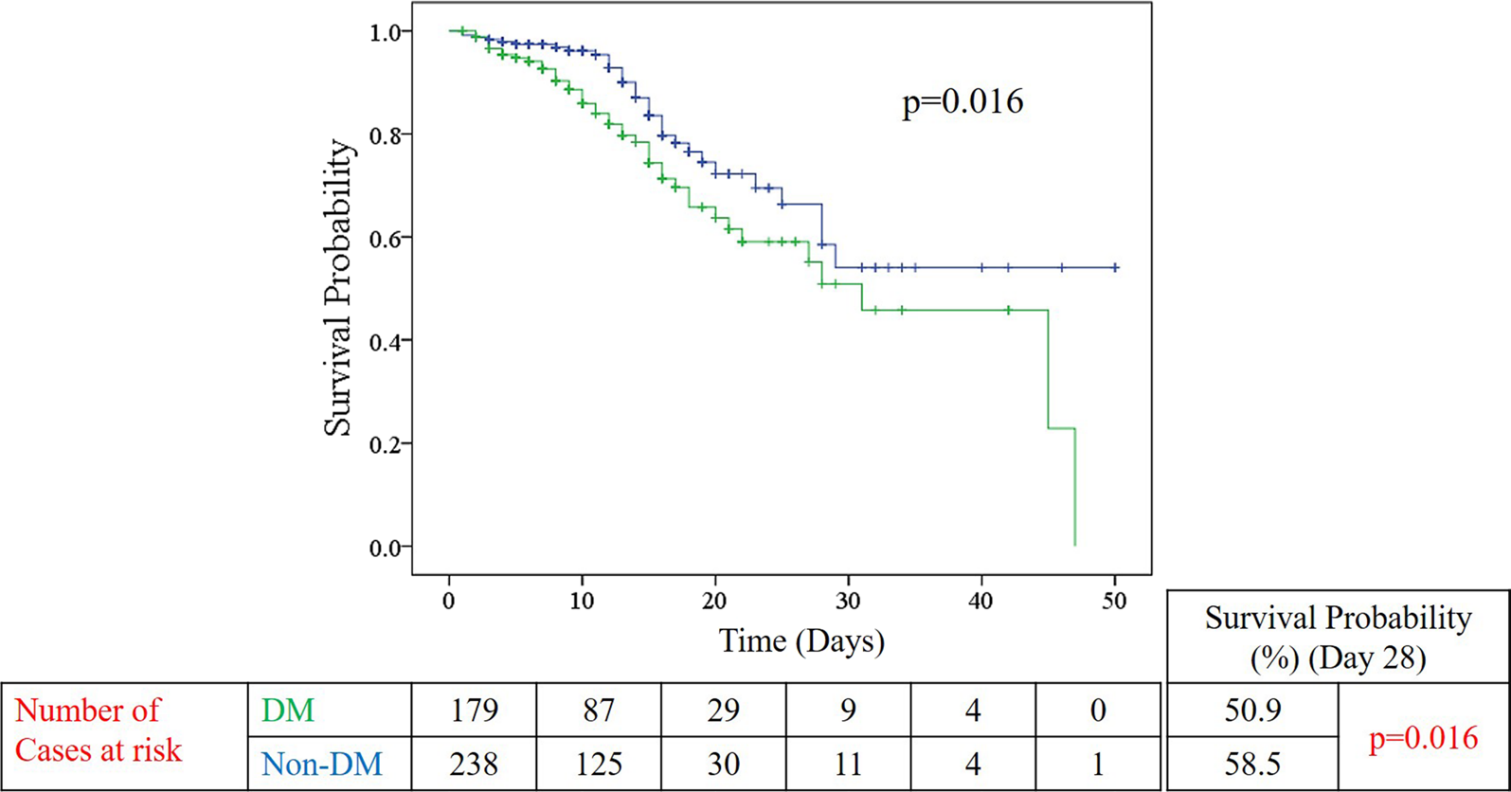
Kaplan–Meier survival analysis according to DM status (DM-green; non-DM-blue). p-value obtained from Log Rank Mantel–Cox test

With regards to DM status, a significantly higher proportion of DM patients received oxygen on arrival to ER (p = 0.006) and were more commonly treated with antibiotics and dexamethasone (p-values < 0.001 and < 0.001, respectively) than non-DM patients. DM patients were also more likely to be intubated than non-DM patients (p = 0.04). Mortality rate in the DM group was significantly higher than the non-DM group (20.5% versus 12.3%; p = 0.04). Furthermore, Kaplan–Meier survival analysis curve showed that DM patients had a significantly shorter survival time than non-DM patients (p = 0.016) (Fig. 2). No differences were observed in terms of duration from diagnosis to admission, intubation, ICU admission, discharge and mortality. The rest of the outcomes were mentioned in Table 4.

A sub-analysis was done to determine differences in mortality rates among Covid-19 patients whose HbA1c levels were measured: non-DM (Hba1c < 5.7; N = 25), prediabetes (HbA1c 5.7–6.4; N = 72) and DM (HbA1c ≥ 6.5; N = 227). Death rates for non-DM, prediabetes and DM were 8.0%, 14.7% and 15.5% (p = 0.58). There was no difference in the prevalence of DM (70.6% versus 66.1%) and death rates (18.6% versus 17.3%) between Saudis and non-Saudis (not shown in tables).

### Significant risk factors for outcomes of interest

**Fig. 3:**
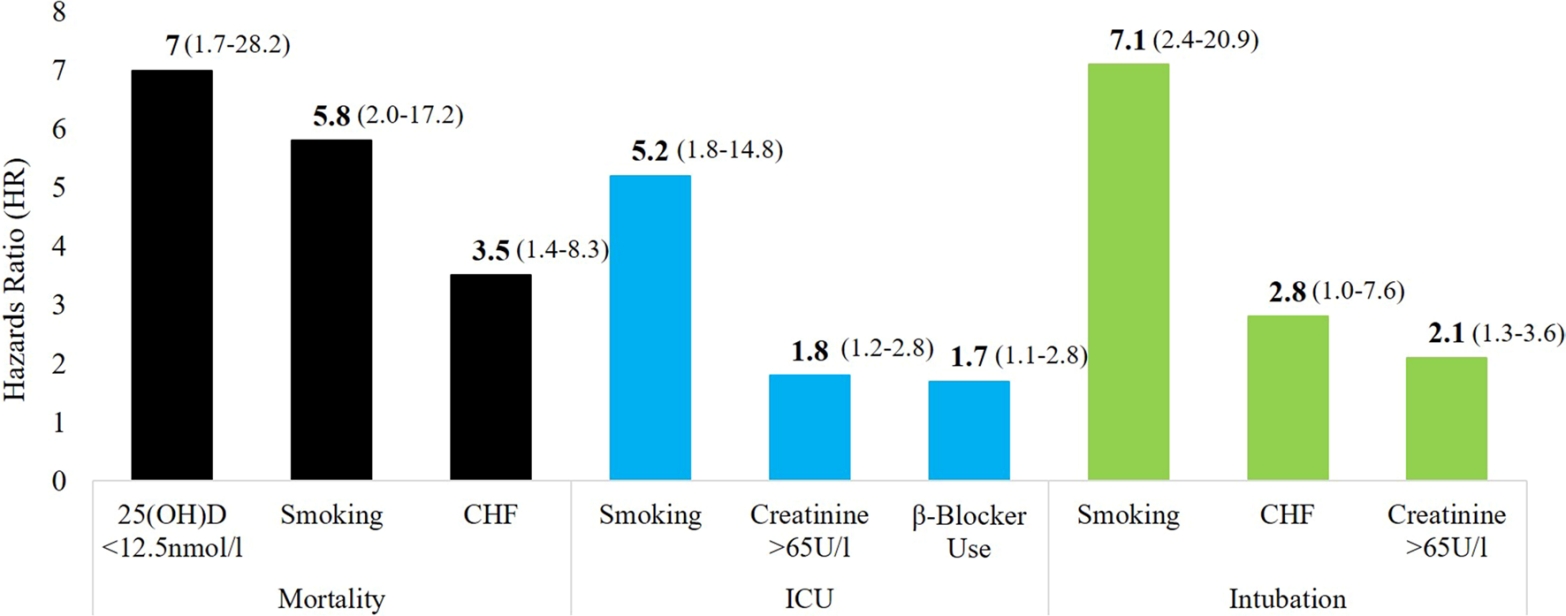
Top significant predictors (adjusted for covariates) [HR (95% CI)] for selected outcomes: mortality (black), ICU (blue) and intubation (green)

Multivariate cox proportional hazards regression model revealed that age [HR 3.0 (CI 1.7–5.3); p < 0.001], congestive heart failure [adjusted HR 3.5 (CI 1.4–8.3); p = 0.006], smoking [adjusted HR 5.8 (CI 2.0–17.2); p < 0.001], β-blocker use [adjusted HR 1.7 (CI 1.0–2.9); p = 0.04], bilateral lung infiltrates [adjusted HR 1.9 (CI 1.1–3.3); p = 0.02], creatinine > 90 µmol/l [adjusted HR 2.1 (CI 1.3–3.5); p = 0.004] and severe vitamin D deficiency [adjusted HR 7.0 (CI 1.7–28.2); p = 0.007] were significant risk factors associated with death among hospitalized Covid-19 patients (Table 5). On the other hand, smoking [adjusted HR 5.2 (CI 1.8–14.8); p = 0.002], β-blocker use [adjusted HR 1.7 (CI 1.1–2.8); p = 0.02], RBG ≥ 11.1 mmol/l [adjusted HR 1.5 (CI 1.0–2.2); p = 0.04], neutrophil count > 7.5 [adjusted HR 1.6 (CI 1.1–2.4); p = 0.02], creatinine > 90 µmol/l [adjusted HR 1.8 (CI 1.2–2.2); p = 0.006] and ALT > 65U/l [adjusted HR 1.6 (CI 1.1–2.4); p = 0.02] were significantly associated with ICU admission. Lastly, the significant risk factors for intubation were congestive heart failure [adjusted HR 2.8 (CI 1.0–7.6); p = 0.048], smoking [adjusted HR 7.1 (CI 2.4–20.9); p < 0.001] and creatinine > 90 µmol/l [adjusted HR 2.1 (CI 1.3–3.6); p = 0.003] (Table 5). Unadjusted HRs with 95% CIs for the parameters mentioned, including inflammatory markers are provided as a Additional file 3. The top predictors for each outcome have been plotted in Fig. 3.

**Table 5:** Factors for outcomes of interest using the multivariate Cox proportional hazards regression model

Risk factor	Outcomes								
	Mortality			ICU admission			Intubation		
	HR	95% CI	p-value	HR	95% CI	p-value	HR	95% CI	p-value
Age > 55 years	3.0	1.7–5.3	< 0.001	1.1	0.7–1.7	0.62	1.4	0.9–2.4	0.14
Male	1.4	0.9–2.4	0.17	1.2	0.8–1.8	0.44	1.4	0.8–2.4	0.22
Comorbidities									
Obesity	1.0	0.6–1.6	0.93	1.2	0.8–1.7	0.47	1.1	0.7–1.8	0.73
Hypertension	0.8	0.4–1.6	0.55	1.0	0.5–1.9	0.97	1.0	0.5–2.1	0.92
Diabetes mellitus	1.2	0.7–2.3	0.49	1.0	0.6–1.7	0.96	1.2	0.6–2.2	0.60
Cardiovascular disease	1.8	0.7–4.4	0.23	1.2	0.5–3.1	0.64	1.0	0.3–3.0	0.96
Chronic kidney disease	0.7	0.2–2.0	0.49	1.0	0.4–2.5	0.95	0.7	0.2–2.5	0.61
Congestive heart failure	3.5	1.4–8.3	0.006	1.9	0.7–5.1	0.19	2.8	1.0–7.6	0.048
Stroke	1.3	0.5–3.8	0.61	1.0	0.4–3.0	0.95	1.9	0.7–5.2	0.20
Smoking	5.8	2.0–17.2	0.002	5.2	1.8–14.8	0.002	7.1	2.4–20.9	< 0.001
Medications									
β-Blocker use	1.7	1.0–2.9	0.04	1.7	1.1–2.8	0.02	1.6	0.9–2.8	0.11
ACE inhibitor use	0.7	0.3–1.4	0.29	1.0	0.6–1.8	0.86	0.9	0.4–1.7	0.66
ARB Use	1.3	0.6–2.6	0.46	1.7	0.8–3.5	0.14	1.4	0.6–3.3	0.38
Laboratory investigations									
RBG (≥ 11.1 mmol/l)	1.2	0.7–1.9	0.54	1.5	1.0–2.2	0.04	1.6	1.0–2.5	0.07
FPG (≥ 7.0 mmol/l)	1.2	0.7–2.2	0.53	1.0	0.6–1.6	0.90	1.1	0.6–1.9	0.81
HbA1c > 9.0%	0.7	0.3–1.3	0.25	1.0	0.6–1.6	0.98	0.9	0.5–1.8	0.85
Bilateral lung infiltrates	1.9	1.1–3.3	0.02	1.4	0.9–2.1	0.14	2.0	1.1–3.4	0.017
Neutrophil count > 7.5	1.4	0.9–2.2	0.19	1.6	1.1–2.4	0.02	1.5	0.9–2.5	0.09
Creatinine > 90 µmol/l	2.1	1.3–3.5	0.004	1.8	1.2–2.8	0.006	2.1	1.3–3.6	0.003
ALT > 65 U/l	1.3	0.8–2.1	0.35	1.6	1.1–2.4	0.02	1.4	0.9–2.4	0.17
25(OH)D < 12.5 nmol/l	7.0	1.7–28.2	0.007	3.0	0.7–13.4	0.16	2.0	0.4–10.1	0.39

Comorbidities adjusted for one another + age, BMI and sex; Medications and Lab investigations were adjusted for age, BMI and Sex; p < 0.05 considered significant

## Discussion

Data from hospitalized Covid-19 patients with outcomes in the Middle East and the Gulf Cooperation Council (GCC) countries in particular, are relatively scarce. To the best of our knowledge, this retrospective study is one of the few, if not the first study to comprehensively present the clinical characteristics and outcomes of Covid-19 patients with and without DM admitted at a tertiary hospital in KSA.

### Main findings

Hospitalized Covid-19 patients with DM outnumber those without by 2:1. Death rate was also significantly higher in the DM than non-DM group, with DM patients generally having worse clinical symptoms and metabolic profile than their non-DM counterparts. These findings largely support previous observations from pooled analyses associating DM Covid-19 severity and outcomes [20, 21]. However, it is noteworthy that DM was not associated with mortality after adjustments for age, sex, BMI, and other pre-existing conditions. Levels of HbA1c, fasting and random glucose on admission were also not associated with mortality after adjusting for covariates. This indicates that DM alone may not fully explain the adverse outcomes associated with Covid-19. Similarly, data from a large scale, multi-center, retrospective study in Wuhan involving 1561 Covid-19 patients described the presence of DM as not independently associated with in-hospital deaths [22]. A more recent evidence in the US involving 463 Covid-19 also support the lack of association between DM and mortality, as well as risk for ICU admission and mechanical ventilation [23]. In contrast, preliminary data in UK indicated that an overwhelming one-third of Covid-19 patients dying in hospitals had DM [24]. The absence of significant risk in mortality observed in the present study does not supersede the fact that DM remains a major risk factor for poor prognosis. It however suggests that the increased risk for worse outcomes is the cumulative effect of DM clustering with other chronic diseases, or cardiometabolic multi-morbidity, which precipitates complications [25, 26].

### Established predictors and potential factors influencing Covid-19 outcomes

The present study identified smoking and elevated creatinine as significant predictors in all outcomes of interest (mortality, ICU admission and intubation). Established risk factors such as increasing age, congestive heart failure, bilateral lung infiltrates, high neutrophil count, hyperglycemia and abnormal ALT were also noted to be independent risk factors. Most of these factors have also been linked, in varying degrees, to the progression of acute respiratory distress syndrome (ARDS) secondary to SARS-CoV and MERS-CoV [27]. Together with DM, all these risk factors contribute to the exacerbation of pre-existing chronic inflammation, progressing to cytokine storm and rapid impairment of endothelial function, if left untreated [28]. The low prevalence of smoking in the study (2.6%) is in alignment with several retrospective observational studies that highlight the unexpectedly low prevalence of smokers among hospitalized patients with Covid-19, but with worse outcomes [29, 30].

Other significant factors for poor outcomes identified in the present study are the use of β-blockers and severe vitamin D deficiency among admitted Covid-19 patients. Both ACE inhibitor and ARB did not appear to significantly alter the outcomes of interest. One theory is that β-blockers can be beneficial, albeit controversial, by reducing pulmonary vascular flow, eventually decreasing further damage to the injured lung, among those suspected of ARDS, a common complication among ICU admitted patients [31]. On the other hand, low vitamin D status has been consistently linked to increased risk of pneumonia and upper respiratory tract infections, secondary to weakened immune system and elevated inflammatory cytokines [32]. Accumulating evidence also link vitamin D deficiency to poor Covid-19 prognosis and mortality [33, 34]. Despite the lack of sufficient data on the use of vitamin D for the prevention and treatment of Covid-19, it has nevertheless been advocated as an adjuvant therapy [35]. Given that vitamin D deficiency is very common in the Arab region, especially among the youth [36], further investigations on the effects of vitamin D supplementation on outcomes of Covid-19 patients are warranted.

### Gender differences in Covid-19 characteristics

In the present study, men outnumber women in hospital admissions by 2:1. Even if non-Saudis are excluded, more men are still getting hospitalized than women. Men also had a higher prevalence of fever and diarrhea, as well as an over-all worse metabolic profile than women, despite having no differences in clinical outcomes. In contrast, most sexual differences among Covid-19 patients conducted elsewhere highlight similar prevalence in men and women, but with a consistently overwhelming worse outcomes in men in terms of morbidity and mortality, establishing male sex as an independent risk factor for Covid-19 [37–39]. The stronger innate and adaptive immune response in females can be attributed to many factors, but mostly to estrogen being immune boosting as opposed to testosterone, being immune suppressing [40]. The disproportionate number of male hospitalizations in the present study can be attributed to several factors, including the higher number of men in the Saudi population over-all and a higher prevalence of male expatriate residents than females. Another factor is that men in KSA and the Arab region over-all, are more socially mobile, more likely to be employed and not as culturally restricted as their female counterparts, putting them at higher risk for Covid-19, if precautions are ignored.

### Strengths and limitations

The authors acknowledge several limitations. Selection bias is imminent and findings are limited to the accuracy of record keeping, given its retrospective design. The correctness of overlapping comorbidities therefore such as CVD and CHF cannot be established other then what is available in the checklist of medical history and medications taken based on his/her account and/or hospital record. The high prevalence of DM in the present study should be interpreted with caution, since the population is not homogenous, and a considerable number of non-Arabs, Indians in particular, who have a different prevalence of DM than Arabs, were included in the analysis. This is important since diabetes and Covid-19 disproportionately affect racial minorities as mentioned previously [17, 18]. Biochemical parameters such as 25(OH)D, to name a few, were absent in most patients and this can affect the findings in terms of decreased power size. Emerging biomarkers in the context of DM and Covid-19 management which are not routinely assessed such as N-terminal–pro-Brain Natriuretic peptide, hs-Troponin and TyG index were not measured and may have added clinical value in the study [41, 42]. Furthermore, duration of DM was not noted and the lack of COPD cases may affect the findings, given that these are major confounding variables. Despite the limitations, the findings of the present study are robust, and adds value to the limited literature on Covid-19 patients within the Arab region, as it is the first to comprehensively describe hospitalized patients and differentiates characteristics based on sex, DM status, severity and outcome.

## Conclusions

In summary, the prevalence of DM is high among hospitalized Covid-19 patients in Riyadh, Saudi Arabia. While DM patients have a higher mortality rate than non-DM patients, other factors such as old age, congestive heart failure, smoking, β-blocker use, the presence of bilateral lung infiltrates, elevated creatinine and severe vitamin D deficiency, appear to be more significant predictors of mortality. Covid-19 patients with either elevated RBG or other acute metabolic impairments (neutrophilia, acute renal and liver dysfunctions) on admission are more likely to receive intensive care. Larger epidemiologic studies covering multiple institutions are needed to determine a more accurate in-hospital death rate in the country.

## Supplementary information

**Additional file 1: Table S1**. Presenting symptoms and vital signs of Covid-19 patients according to severity and final outcome.

**Additional file 2: Table S2**. Clinical characteristics of Covid-19 patients according to severity and outcome.

**Additional file 3: Table S3**. Factors for outcomes of interest using the multivariate Cox proportional hazards regression model.

## Data Availability

The datasets used and/or analyzed during the current study are available from the corresponding author on reasonable request.

## Abbreviations

ACE: Angiotensin converting enzyme
ALT: Alanine transferase
ARB: Angiotensin receptor blockers
ARDS: Acute respiratory distress syndrome
AST: Aspartate aminotransferase
BMI: Body mass index
BUN: Blood urea nitrogen
Ca: Calcium
CCB: Calcium channel blockers
CI: Confidence interval
CT: Cycle threshold
Covid-19: Coronavirus disease-19
CRP: C-reactive protein
DM: Diabetes mellitus
ER: Emergency room
ESR: Erythrocyte sedimentation rate
FBG: Fasting blood sugar
FT4: Free thyroxine
GLP-1: Glucagon-like peptide-1
HbA1c: Glycated hemoglobin
HDL: High density lipoprotein
HR: Hazards ratio
ICU: Intensive care unit
IL-6: Interleukin-6
IRB: Institutional Review Board
K: Potassium
KSA: Kingdom of Saudi Arabia
KKUH: King Khalid University Hospital
KSUMC: King Khaled University Medical City
LDH: Lactate dehydrogenase
LDL: Low density lipoprotein
MERS-CoV: Middle East Respiratory Syndrome coronavirus
Na: Sodium
NS: Not significant
RT-PCR: Reverse transcription polymerase chain reaction
RBG: Random blood sugar
SARS-CoV: Severe acute respiratory syndrome coronavirus
SARS-CoV2: Severe acute respiratory syndrome coronavirus 2
SpO2: Oxygen saturation
TSH: Thyroid stimulating hormone
VDD: Vitamin D deficiency
WBC: White blood count
