# Supplementary material for: Diabetes and Covid-19 among hospitalized patients in Saudi Arabia: a single-centre retrospective study
Source: Cardiovasc Diabetol. 2020 Dec 5;19:205. doi: 10.1186/s12933-020-01184-4 (PMC7718833; doi:10.1186/s12933-020-01184-4)
Supplement: Supplementary file 1 — Additional file 1: Table S1. Presenting symptoms and vital signs of Covid-19 patients according to severity and final outcome. [file 12933_2020_1184_MOESM1_ESM.docx]

**Table S1**. Presenting Symptoms and Vital Signs of Covid-19 Patients according to Severity and Final Outcome.

| **Parameters** | **SEVERITY** | | **FINAL OUTCOME** | |
| --- | --- | --- | --- | --- |
|  | Non-Severe | Severe | Discharged | Died |
| N | 316 | 123 | 343 | 77 |
| **Symptoms** | | | | |
| Fever (%) | 232 (75.1) | 95 (80.5) | 261 (75.4) | 61 (80.3) |
| Cough (%) | 214 (68.8) | 87 (73.7) | 244 (70.1) | 53 (69.7) |
| Dyspnea (%) | 223 (71.7) | 90 (75.6) | 247 (71.0) | 62 (80.5) |
| Nausea/Vomiting (%) | 75 (24.1) | 24 (20.3) | 84 (24.1) | 13 (17.1) |
| Diarrhea (%) | 62 (19.9) | 29 (24.8) | 77 (22.1) | 13 (17.3) |
| Anosmia (%) | 16 (5.1) | 4 (3.4) | 18 (5.2) | 1 (1.3) |
| Ageusia (%) | 17(5.5) | 6 (5.0) | 19 (5.4) | 3 (3.9) |
| Myalgia (%) | 32 (10.3) | 7 (5.9) | 36 (10.3) | 2 (2.7) |
| **Vital Signs*** | | | | |
| Temperature (°C) | 37.6 ± 0.9 | 37.6 ± 0.8 | 37.6 ± 0.8 | 37.7 ± 0.9 |
| Heart Rate (beats/minute) | 96.4 ± 17.1 | 97.1 ± 19.9 | 96.7 ± 17.6 | 95.6 ± 19.8 |
| Respiratory Rate (breaths/min) | 27.6 ± 7.7 | 24.5 ± 9.3* | 24.8 ± 8.9 | 27.9 ± 8.8* |
| Systolic Blood Pressure (mmHg) | 123.7 ± 17.8 | 128.1 ± 24.2 | 125.2 ± 19.3 | 123.3 ± 22.9 |
| Diastolic Blood Pressure (mmHg) | 72.2 ± 13.9 | 73.6 ± 13.0 | 74.2 ± 12.6 | 68.4 ± 14.3* |
| SpO_2_ (%) | 92.2 ± 7.6 | 88.2 ± 10.5* | 92.0 ± 7.5 | 87.3 ± 12.1* |

**Note:** SpO_2_, oxygen saturation; *****Bonferroni adjusted p-value for multiple comparisons, significant at **p<0.008**.
