# Supplementary material for: Diabetes and Covid-19 among hospitalized patients in Saudi Arabia: a single-centre retrospective study
Source: Cardiovasc Diabetol. 2020 Dec 5;19:205. doi: 10.1186/s12933-020-01184-4 (PMC7718833; doi:10.1186/s12933-020-01184-4)
Supplement: Supplementary file 2 — Additional file 2: Table S2. Clinical characteristics of Covid-19 patients according to severity and outcome. [file 12933_2020_1184_MOESM2_ESM.docx]

**Table S2**. Clinical Characteristics of Covid-19 Patients according to Severity and Outcome.

| **Parameters** | **SEVERITY** | | **OUTCOME** | |
| --- | --- | --- | --- | --- |
|  | Non-Severe | Severe | Discharged | Died |
| N | 316 | 123 | 343 | 77 |
| **Chest X-Ray**  No infiltrates  Unilateral infiltrates  **Bilateral lung infiltrates** | 108 (34.6)  28 (9.0)  **170 (54.5)** | 22 (18.2)  14 (11.6)  **85 (70.2)*** | 117 (34.1)  39 (11.4)  **187 (54.5)** | 14 (18.7)  3 (4.0)  **58 (77.3)*** |
| **Complete Blood Count** | | | | |
| Hemoglobin (g/l) (120-160) | 130.8 ± 1.4 | 128.6 ± 1.7 | 132.4 ± 1.2 | 120.8 ± 3.0* |
| WBC count (4.0-11.0) | 7.6 ± 0.2 | 8.7 ± 0.4 | 7.8 ± 0.2 | 8.8 ± 0.7 |
| Platelet count (140-450) | 253.6 ± 5.8 | 241.2 ± 8.1 | 253.6 ± 5.4 | 236.6 ± 10.4 |
| Lymphocyte (1-5) | 1.4 ± 0.1 | 1.0 ± 0.08 | 1.3 ± 0.07 | 1.4 ± 0.4 |
| Neutrophils (2.0-7.5) | 5.6 ± 0.2 | 7.0 ± 0.4 | 5.7 ± 0.2 | 7.6 ± 0.8* |
| **D-Dimer (µg/ml) (0.22-0.45)** | **1.8 ± 0.2** | **3.3 ± 0.4*** | **1.9 ± 0.2** | **3.4 ± 0.5*** |
| **Liver Profile** | | | | |
| ALT (U/l) (20-65) | 54.1 ± 3.5 | 65.6 ± 6.4 | 58.1 ± 3.6 | 58.7 ± 6.8 |
| AST (U/l) (15-37) | 52.9 ± 3.0 | 76.6 ± 6.6* | 58.7 ± 3.5 | 70.8 ± 6.5 |
| LDH (U/l) (84-246) | 416.0 ± 12.7 | 546.2 ± 25.3* | 438.1 ± 13.2 | 533.4 ± 28.9 |
| **Renal Profile** | | | | |
| BUN (mmol/l) (2.5-6.4) | 7.0 ± 0.4 | 9.5 ± 0.9 | 6.6 ± 0.4 | 12.8 ± 1.5* |
| Creatinine (µmol/l) (49-90) | 110.5 ± 8.4 | 153.4 ± 18.1 | 105.8 ± 7.0 | 202.5 ± 30.2* |
| Na (mmol/l) (136-145) | 137.0 ± 0.3 | 136.3 ± 0.6 | 136.8 ± 0.3 | 137.1 ± 0.8 |
| K (mmol/l) (3.5-5.1) | 4.4 ± 0.1 | 4.5 ± 0.09 | 4.3 ± 0.03 | 5.2 ± 0.5* |
| **Lipid Profile** | | | | |
| Triglycerides (mmol/l) | 1.8 ± 0.1 | 2.4 ± 0.2 | 1.9 ± 0.08 | 2.6 ± 0.3* |
| HDL-Cholesterol (mmol/l) | 1.0 ± 0.08 | 0.77 ± 0.04 | 0.94 ± 0.06 | 0.71 ± 0.05 |
| LDL-Cholesterol (mmol/l) | 2.2 ± 0.1 | 1.8 ± 0.1 | 2.2 ± 0.1 | 1.6 ± 0.2 |
| **Inflammatory Markers** | | | | |
| **Ferritin (µg/ml) (13-150)** | **825 ± 52.8** | **1243 ± 137*** | **841 ± 47.8** | **1457 ± 214*** |
| Procalcitonin (ng/ml) (0-0.046) | 1.5 ± 0.6 | 4.5 ± 1.4 | 2.0 ± 0.7 | 4.5 ± 1.4 |
| ESR (mm/hr) (0-24) | 67.0 ± 2.4 | 76.9 ± 3.1 | 67.8 ± 2.2 | 81.6 ± 3.9 |
| **CRP (mg/l) (<10.0)** | **96.6 ± 4.2** | **124.1 ± 7.6*** | **95.5 ± 3.8** | **148.1 ± 10.7*** |
| IL-6 (pg/ml) (1.5-7.0) | 175.1 ± 43.1 | 170.5 ± 30.9 | 167.1 ± 35.5 | 189.3 ± 42.6 |
| **Thyroid Profile** | | | | |
| TSH (µIU/ml) (0.25-5.0) | 2.5 ± 0.7 | 1.1 ± 0.2 | 2.2 ± 0.6 | 1.7 ± 0.4 |
| FT4 (pmol/l) (10-24.5) | 16.4 ± 0.3 | 16.6 ± 0.6 | 16.9 ± 0.3 | 14.6 ± 0.6 |
| **Glycemic Profile** | | | | |
| HbA1c (%) | 7.9 ± 0.2 | 8.4 ± 0.3 | 8.0 ± 0.2 | 8.1 ± 0.3 |
| Fasting Glucose (mmol/l) | 8.9 ± 0.3 | 10.0 ± 0.5 | 8.9 ± 0.3 | 10.8 ± 0.8 |
| **Other Markers** | | | | |
| Corrected Ca (mmol/l) (2.1-2.55) | 2.3 ± 0.2 | 2.3 ± 0.2 | 2.3 ± 0.01 | 2.3 ± 0.02 |
| 25(OH)D (nmol/l) (75-250) | 40.9 ± 3.1 | 40.3 ± 3.0 | 49.9 ± 8.8 | 39.0 ± 2.4 |

**Note:** WBC, white blood count; ALT, alanine transferase; AST, aspartate aminotransferase; LDH, lactate dehydrogenase; BUN, blood urea nitrogen; Na, sodium; K, potassium; HDL, high density lipoprotein; LDL, low density lipoprotein; ESR, erythrocyte sedimentation rate; CRP, C-reactive protein; IL-6, interleukin-6; TSH, thyroid stimulating hormone; FT4, free thyroxine; HbA1c, glycated hemoglobin; Ca, calcium; *denotes significance at **p<0.0018**.
