# Supplementary material for: Diabetes and Covid-19 among hospitalized patients in Saudi Arabia: a single-centre retrospective study
Source: Cardiovasc Diabetol. 2020 Dec 5;19:205. doi: 10.1186/s12933-020-01184-4 (PMC7718833; doi:10.1186/s12933-020-01184-4)
Supplement: Supplementary file 3 — Additional file 3: Table S3. Factors for outcomes of interest using the multivariate Cox proportional hazards regression model. [file 12933_2020_1184_MOESM3_ESM.docx]

**Table S3**. Factors for Outcomes of Interest Using the Multivariate Cox Proportional Hazards Regression Model **(UNADJUSTED)**.

| **Risk Factor** | **Outcomes** | | | | | | | | |
| --- | --- | --- | --- | --- | --- | --- | --- | --- | --- |
|  | **Mortality** | | | **ICU Admission** | | | **Intubation** | | |
|  | **HR** | **95% CI** | **P-Value** | **HR** | **95% CI** | **P-Value** | **HR** | **95% CI** | **P-Value** |
| Age > 55 years | 1.6 | 1.3-2.0 | <0.001 | 1.2 | 0.8-1.7 | 0.36 | 1.4 | 0.9-2.3 | 0.14 |
| Male | 1.2 | 0.7-2.0 | 0.50 | 1.2 | 0.8-1.8 | 0.28 | 0.98 | 0.8-1.2 | 0.89 |
| **Comorbidities** | | | | | | | | | |
| Obesity | 1.1 | 0.7-1.8 | 0.58 | 1.1 | 0.8-1.6 | 0.45 | 1.0 | 0.8-1.3 | 0.66 |
| Hypertension | 0.6 | 0.4-0.9 | 0.02 | 0.9 | 0.6-1.2 | 0.40 | 0.89 | 0.6-1.4 | 0.63 |
| Diabetes Mellitus | 1.7 | 1.1-2.7 | 0.018 | 0.9 | 0.7-1.3 | 0.75 | 1.0 | 0.6-1.6 | 0.88 |
| Cardiovascular Disease | 1.0 | 0.7-1.5 | 0.78 | 1.2 | 0.7-2.2 | 0.45 | 0.9 | 0.6-1.3 | 0.70 |
| Chronic Kidney Disease | 1.7 | 0.99-3.0 | 0.053 | 1.6 | 0.7-3.1 | 0.27 | 0.9 | 0.6-1.5 | 0.74 |
| Congestive Heart Failure | 1.6 | 0.8-3.1 | 0.17 | 1.7 | 0.8-3.6 | 0.15 | 1.1 | 0.5-2.3 | 0.89 |
| Stroke | 1.6 | 0.8-3.0 | 0.18 | 1.2 | 0.5-2.8 | 0.74 | 1.4 | 0.6-3.2 | 0.43 |
| Smoking | 1.0 | 0.4-2.8 | 0.94 | 1.7 | 0.7-4.2 | 0.26 | 1.6 | 0.6-4.5 | 0.37 |
| **Medications** | | | | | | | | | |
| β-Blocker Use | 1.2 | 0.9-1.7 | 0.20 | 1.1 | 0.7-1.7 | 0.67 | 0.9 | 0.5-1.5 | 0.61 |
| ACE Inhibitor Use | 1.1 | 0.6-2.3 | 0.71 | 1.2 | 0.7-2.0 | 0.52 | 0.97 | 0.5-2.0 | 0.94 |
| ARB Use | 0.9 | 0.5-1.7 | 0.70 | 1.1 | 0.6-2.1 | 0.68 | 1.1 | 0.8-1.4 | 0.68 |
| **Laboratory Investigations** | | | | | | | | | |
| RBG (≥11.1mmol/l) | 1.1 | 0.9-1.4 | 0.41 | 1.1 | 0.8-1.6 | 0.50 | 1.1 | 0.8-1.4 | 0.60 |
| FPG (≥7.0mmol/l) | 1.5 | 0.8-2.6 | 0.19 | 1.2 | 0.8-1.8 | 0.47 | 1.1 | 0.9-1.4 | 0.38 |
| HbA1c > 9.0 % | 0.86 | 0.6-1.1 | 0.26 | 1.1 | 0.7-1.8 | 0.57 | 1.1 | 0.8-1.4 | 0.59 |
| Bilateral Lung Infiltrates | 2.1 | 1.2-3.6 | 0.007 | 1.1 | 0.7-1.6 | 0.63 | 1.2 | 0.9-1.4 | 0.19 |
| Neutrophil Count >7.5 | 1.5 | 1.2-2.0 | 0.002 | 0.8 | 0.5-1.1 | 0.16 | 1.2 | 0.9-1.5 | 0.20 |
| Creatinine > 90µmol/l | 2.2 | 1.4-3.5 | 0.001 | 1.1 | 0.8-1.5 | 0.68 | 1.2 | 0.96-1.5 | 0.11 |
| ALT > 65 U/l | 1.1 | 0.6-1.8 | 0.82 | 0.64 | 0.4-0.9 | 0.03 | 1.1 | 0.8-1.4 | 0.60 |
| 25(OH)D <12.5nmol/l | 2.1 | 0.8-5.6 | 0.14 | 0.3 | 0.07-1.4 | 0.13 | 1.2 | 0.5-2.9 | 0.73 |
| **Inflammatory Markers** |  |  |  |  |  |  |  |  |  |
| D-Dimer (µg/ml) # | 2.2 | 1.4-3.5 | 0.001 | 1.5 | 0.99-1.1 | 0.07 | 2.0 | 1.2-3.6 | 0.01 |
| Ferritin (µg/ml) # | 1.4 | 0.9-2.2 | 0.14 | 1.1 | 0.8-1.6 | 0.47 | 1.4 | 0.9-2.3 | 0.14 |
| Procalcitonin (ng/ml) # | 1.6 | 1.2-2.0 | <0.001 | 1.2 | 0.97-1.5 | 0.09 | 1.3 | 0.98-1.8 | 0.07 |
| ESR (mm/hr) # | 3.2 | 0.9-11.2 | 0.07 | 1.2 | 0.6-2.4 | 0.64 | 0.99 | 0.3-3.2 | 0.98 |
| CRP (mg/l) # | 2.6 | 1.4-5.0 | 0.004 | 1.2 | 0.8-1.8 | 0.39 | 1.1 | 0.6-2.0 | 0.85 |
| IL-6 (pg/ml) # | 1.6 | 0.98-2.6 | 0.06 | 1.0 | 0.7-1.4 | 0.86 | 1.1 | 0.7-1.6 | 0.82 |

**Note:** # denotes log-transformed variables; P<0.05 considered significant.
